# The past and future of breast cancer treatment—from the papyrus to individualised treatment approaches

**DOI:** 10.3332/ecancer.2017.746

**Published:** 2017-06-08

**Authors:** Felipe Ades, Konstantinos Tryfonidis, Dimitrios Zardavas

**Affiliations:** 1 Hospital Albert Einstein, Avenida Albert Einstein, 627 - Morumbi, São Paulo - SP, 05652-900 Brazil; 2 European Organisation for Research and Treatment of Cancer, Avenue E. Mounier 83/11, 1200 Brussels, Belgium; 3 Breast International Group (BIG), Boulevard de Waterloo 76, Brussels 1000, Belgium

**Keywords:** breast cancer, cancer history, breast surgery, gene signature, molecular profiling, liquid biopsy

## Abstract

Cancer is one of the oldest diseases ever described, since ancient Egypt there have always been attempts to treat and cure this illness. The growing body of knowledge about breast cancer biology and improvements in surgical and medical treatments has been built over time with contributions from many talented and enthusiastic physicians and researchers. Medical advances have changed the approach from a previously incurable condition, into a surgical disease. Further improvements in cancer biology have allowed the development of systemic treatments, hormonal therapies, and targeted drugs. The description of the molecular intrinsic subtypes of breast cancer clarified the understanding of breast cancer as a group of heterogeneous diseases, associated with different clinical outcomes, and therapeutic opportunities. This paper reviews how breast cancer treatment has improved since the earliest descriptions, in ancient times, and how future approaches, such as gene signatures, molecular profiling, and liquid biopsies, aim to further develop individualised treatments and improve treatment outcomes.

## Breast cancer treatment in ancient times

Cancer is one of the oldest diseases ever described by medicine. One of the earliest manuscripts reporting on cancer treatment was the Egyptian papyrus of Edwin Smith, dated 1600BC but possibly a copy from a much older document, from 2500 to 3000BC [1]. It is considered to be the first known medical treatise, as it embraces a rational and scientific approach towards medical treatments, in contrast with older manuscripts that reported magic and other mystical methods to treat diseases. The papyrus describes 48 generic clinical cases where rudimentary surgical procedures were used to treat wounds, fractures, and cancer, according to the different parts of the body. The part dedicated to the breast depicts techniques to treat breast cancer and wounds, but not with the objective to eradicate with curative intent, as cancer, at that time, was considered an incurable illness [2].

Around the year 400BC, the Greek physician Hyppocrates, considered the ‘father of medicine’, created the term cancer. The name derived from the word ‘karkinos’, which is the Greek term for crab or crayfish, in an analogy for the invasive behaviour of the disease, touching and invading nearby tissues [3]. Others believe that the analogy was due to the similarities between the vascularisation of the tumour and the crayfish’s legs. In the second century, the also Greek doctor Galen created the term oncos (the Greek work for swelling) to refer to malignant diseases. Galen believed that diseases were caused by an imbalance of humours and breast cancer had a systemic nature due to the accumulation of black bile in the blood [4].

## From the middle ages to the present day

With the ascension of the monotheistic religions, little progress in medicine was made in Europe. Early Christian views attributed the cause of diseases to God, and treatments, likewise, were based on faith and miracles rather than on surgery and medications. Only in the 10th century, with the rise of the Islamic empire, was Greek medicine revived and expanded due to the work of physicians like Ibn Sina [5] and Abu Al-Qasim Al-Zahrawi (aka Albucasis) [6]. Accurate translations of the medical manuscripts and the establishment of the early medical schools allowed the continuation of medical development [7]. However, the dissection and representation of the human body remained forbidden by religious traditions, limiting additional expansion of medical knowledge [8].

Albucasis, along with the French surgeons Henri de Mondeville [9] and Guy de Chayliac [10], added unique instruments to the surgical procedures to remove breast tumours. But it was not until the 16th century that breast surgery flourished. The improvement of surgical and medical sciences occurred in the context of the age of enlightenment. Detailed anatomical descriptions made by Adreas Versalius, Leonardo da Vinci and others paved the road to a better understanding of the human body allowing the advance of surgical techniques [8]. In Scotland, the surgeon John Hunter [11] established the initial concept of staging; ‘if the tumour is moveable, there is no impropriety in removing it’ [12]. Nevertheless, surgical approaches were limited due to technology issues; until the mid-19th century, anaesthesia was not yet developed for surgical procedures [13]. Surgeons had to rely on technique and, mostly, speed to perform tumour resections. In parallel, investigations on a wide range of carcinogens suspected to be involved in breast cancer development and progression started to be conducted [7]. For the first time, medicine aimed to cure breast cancer.

## 19th century—the beginning of modern breast cancer treatment

The 19th century saw great improvements in the understanding of the disease and its mechanisms. Observations made by Virchow [14] and others underpinned the cellular nature of cancer cells and its differences from their healthy counterparts. This observation dismissed many of the previous humoural theories as causes of cancer and its metastasis. Metastatic spread was due to the dissemination of these cells by the lymphatic and blood vessels.

A range of new surgical approaches were developed given the growing body of information on carcinogenesis and metastasis mechanisms and important improvements were made on surgical techniques, such as anaesthesia, surgical gloves and vestments, and disinfection.

In particular, anaesthesia [13] and disinfection allowed, for the first time, surgical freedom to perform wider resections. The *en bloc* resection was experimented by a range of skilled surgeons such as Charles Moore [15], in the United Kingdom, and Kuster and Volkmann [16], in Germany. Axillary dissection was used as part of the surgical treatment of breast cancer by Banks in Liverpool [17]. In 1894, the surgeon William Halsted [18] described the surgery that became the standard of care for many years to come, the radical Halsted mastectomy. Believing that the disease had an initial local behaviour with a constant path of dissemination, from the breast to the axillary nodes, and only then to distant locations of the body, Halsted developed a technique that would remove the whole tumour in one piece along with the pectoral muscles, lymphatic vessels, and the axillary lymph nodes [18]. The one piece removal would avoid unattended cancerous tissues being left behind and no lymphatic vessels would be damaged, thus no contamination of healthy tissue by cancer cells would occur [19]. Despite the radical approach and the profound side effects of this wide procedure, this was the first time in history that breast cancer could be systematically and scientifically cured.

## Modern times—more improvements and fewer morbidities

In the 20th century, a rapid expansion in medical knowledge was made, with advances seen in multiple areas of medicine. As with surgery, improvements in radiotherapy, chemotherapy, and endocrine therapy were preceded by an accumulation of knowledge about the pathologic mechanisms of the disease.

The surgeon Thomas Beatson, experimenting with animal models, observed that breast tumours in these animals regressed after oophorectomy [20]. The relation of sexual hormones with breast cancer was only elucidated later, in 1967, when Elwood Jensen described the oestrogen receptor, paving the road for the development of a range of oestrogen-modulating drugs [21].

The development of chemotherapy also happened in the second half of the 20th century; first with the mustard gas derivatives [22], followed by a rapid expansion in the chemotherapy portfolio after the heavy financing of cancer drug research established by the US National Cancer Act of 1971 [23]. The pivotal work of the oncologists Bernard Fisher [24], in the USA, and Gianni Bonadonna [25], in Italy, investigated the role of cytotoxic drugs in improving breast cancer cure, inaugurating the concept of adjuvant treatment. In 1975, Gianni Bonadonna presented the first report on the efficacy of cyclophosphamide, methotrexate, and fluorouracil (CMF) as adjuvant treatment. These results, along with those reported by Dr Fisher´s National Surgical Adjuvant Breast and Bowel Project, raised hopes that chemotherapy could have a major role in the management of breast cancer, and were of seminal importance for all the studies on adjuvant systemic therapy conducted throughout the world [26]. Since then, breast cancer chemotherapy schedules improved from the methotrexate combinations, to anthracyclines and the incorporation of taxanes in the 1990s [27].

Taking advantage of the available new therapeutic options, visionary physicians started to combine treatment modalities, improving results, and reducing morbidities. One of the most preeminent surgeons leading this shift in breast cancer treatment was Umberto Veronesi. Working with the oncologist Gianni Bonadonna, at the Istituto Nazionale Tumori, in Milan, the Italian team aimed to perform partial breast surgeries followed by radiotherapy, chemotherapy, and tamoxifen, when applicable. The concept of sentinel lymph node dissection to avoid full axillary dissection was also investigated in this context. This less aggressive approach was proved to be as effective as the more radical and mutilating Halsted method [28]. Tailoring the surgery method and the adjuvant treatment according to each patient started to be a reality. The era of personalised therapy was inaugurated.

## Molecular era of cancer treatment

The proof of concept demonstrated by treatment tailoring according to each patient’s characteristics, staging, and molecular profile was a watershed in cancer treatment and research. Advances achieved in breast cancer treatment served as models to improvements in many other areas of oncology.

Conventional histological evaluation of breast cancer by pathologists has identified several histological subtypes; however, this morphology-based breast cancer taxonomy is of limited relevance in terms of tailoring treatment strategies for individual patients. More than 15 years ago, the employment of the back then newly developed microarrays and the gene expression profiling analysis techniques resulted in the identification of the so-called breast cancer intrinsic subtypes [29, 30]. This proved to be a major conceptual breakthrough, since it identified different molecular subtypes of breast cancer with distinct clinical behaviour and therapeutic vulnerabilities that can be summarised as follows: (i) luminal A breast cancer with expression profile reminiscent of luminal lineage mammary cells, with indolent clinical behaviour, heightened ER-signalling pathways activation and sensitivity to endocrine treatment, (ii) luminal B breast cancer showing poorer prognosis as compared to their luminal A counterparts and higher proliferation status coupled with reduced endocrine sensitivity [31], (iii) HER2-like, associated with HER2/ErbB2 gene amplification, and (iv) basal-like breast cancer, associated with strong basal epithelial marker expression, as well as hormone receptor negativity, high proliferation levels and reduced luminal epithelial and cytokeratin expression [29].

Treatment and clinical research started to rely not only on morphologic and clinical–pathologic features but on cellular behaviour. Deeper understanding of the cellular and molecular biology of breast cancer allowed the detection of genetic aberrations possibly targetable by new compounds [32].

## Breast cancer treatment, what next?

Breast cancer treatment, as it stands today, is selected according to the ‘group of patients’ a specific individual ‘fits in’. As has happened in the past, we are coming to a moment where different approaches to breast cancer treatment need to be developed. Selecting treatments according to the results of the clinical and available molecular tests, indeed, enriches the chance of response to a given treatment strategy, by classifying the patient to a specific subgroup. Nevertheless, individual characteristics that could play a role in the selected patient remain an open area of research. Understanding the individual molecular patterns and aberrations of each patient is an approach that could change today’s ‘stratified’ treatment, based on the intrinsic subtype groups, to a really individualised treatment, based on patients’ specific molecular characteristic.

The open question is how we take the heritage of knowledge produced by our professors from the past and move on to another level, towards really personalised medicine?

## Molecular dissection of breast cancer: intrinsic subtypes and beyond

During recent years, there has been increasing reference to so-called personalised cancer medicine, defined as: ‘A form of medicine that uses information about a person’s genes, proteins, and environment to prevent, diagnose, and treat disease. In cancer, personalised medicine uses specific information about a person’s tumour to help diagnose, plan treatment, find out how well treatment is working, or make a prognosis’ [33]. Personalised medicine has been alternatively described by the term ‘precision medicine’ and it represents an effort to individualise the clinical practice applied to any given patient [34].

In the field of breast cancer, there was an early implementation of some of the above-mentioned principles of personalised cancer medicine, namely through the therapeutic targeting of hormone receptor signalling [35]. The successful clinical development of tamoxifen for patients with hormone receptor-positive breast cancer represents the archetype of personalised medicine applied in oncology [36]. Subsequently, the development of trastuzumab, the first-in-class HER2-blocking agent, for patients with HER2-positive disease exemplified further the improved clinical outcomes that personalised cancer medicine can achieve [37]. Of note, in both cases, a true personalisation is yet to be achieved; it is more accurate to refer to stratified cancer medicine, given that for individual patients with hormone receptor positive or HER2-positive breast cancer treated with endocrine treatment or HER2 blockade, respectively, therapeutic resistance can occur. This indicates that further refinement of triaging patients with the respective treatment is needed, for implementing truly personalised cancer medicine.

The introduction of the intrinsic subtypes of breast cancer, maybe more than anything else, contributed to the dissemination of a conceptual advance; that of breast cancer being a group of heterogeneous diseases, associated with different clinical outcomes and therapeutic opportunities, as will be detailed later. Both aspects have been capitalised ever since to achieve improved patient stratification in terms of accurate prognostication and treatment decisions, bringing us several steps closer to the realisation of personalised breast cancer medicine.

The distinct nature of the breast cancer intrinsic subtypes has been reinforced by studies that coupled gene expression-profiling analysis with gene copy number analysis, indicating that the former ones are associated with recurrent copy number aberrations (CNA). More recently, the introduction of powerful next-generation sequencing (NGS), also known as massively parallel sequencing, enabled an unprecedentedly detailed characterisation of the molecular underpinnings of breast cancer. Several studies applied this high-throughput molecular analysis method to collections of primary breast tumour samples, identifying recurrent gene mutations and/or CNAs among the different intrinsic subtypes; some of them are currently pursued as potential therapeutic targets (Table 1) [38, 39].

## Improving prognostication of patients with early-stage breast cancer

The advent of microarrays contributed to more accurate breast cancer patient stratification, not only through the identification of the afore-mentioned intrinsic subtypes, but also through the development of the so-called first-generation prognostic gene signatures. These multigene prognosticators have been developed through the assessment of the epithelial compartment of primary breast tumours. Currently, several of these first-generation prognostic gene signatures are clinically available: (i) Endopredict (Sividon Diagnostics, Cologne, Germany) (ii) MammaPrint (Agendia, Amsterdam, the Netherlands), (iii) MapQuant DX (Ispogen, Marseille, France), (iv) Oncotype DX (Genomic Health, CA, USA), (v) PAM50 (Nanostring Technologies, WA, USA), (vi) Theros (bioTheranostics, CA, USA), (v) (Table 2). The importance of such multigene prognostic data is emphasised in the newest AJCC breast cancer staging system 2017 update. In this update, when molecular data is available, it can influence the prognostic classification of the disease, providing additional information to the traditional TNM evaluation [40].

It should be noted that the above mentioned first-generation prognostic gene signatures can refine the prognostication of patients with hormone receptor positive early-stage breast cancer, but not other subtypes of the disease; indeed, the two fundamental biological phenomena assessed through them are ER signalling and proliferation status. Furthermore, they disregard the stromal compartment of the disease, for which there is increasing evidence supporting its functional importance for malignant progression through interactions between cancer and stromal/immune cells [41, 42]. Consecutively, there has been a second wave of efforts trying to further refine the prognostication of patients with operable breast cancer, assessing the functionally important stromal component of the disease, as well as the differences among the distinct molecular subtypes.

This has been exemplified by the development of a stroma-derived prognostic predictor (SDPP), a 26-gene prognosticator that was developed through an assessment of tumour stroma [43]. Another example of a second-generation prognostic gene signature was developed through a comparison of CD10+ cells from cancerous and normal mammary tissues, leading to a 12-gene prognostic signature [44]. Regarding triple-negative breast cancer, there have been initial efforts to generate immune-related prognostic signatures with promising results [45, 46]. More recently, a study mining data from The Cancer Genome Atlas reported two immune/inflammatory gene signatures, one associated with poor and one with favourable prognosis among patients with basal-like breast cancer [47].

It should be noted that there is no prospective evidence for second-generation prognostic gene signatures supporting their implementation in clinical practice from prospectively conducted randomised studies; thus, their clinical utility still needs to be proven [48]. Truly personalised prognostication of patients with early-stage breast cancer is a goal to be further pursued that could be reached through the assessment of plasma-based biomarkers.

## Tailoring systemic treatment

There is an increasing evidence that patient-derived tumour xenografts (PDX) models, alternatively termed tumour avatar models, can be valuable tools promoting cancer translational research and advance personalised cancer medicine [49].

## The promise of liquid biopsies

A conceptual breakthrough has been introduced during recent years in the field of oncology, promising to take us several steps closer to truly personalised cancer medicine: that of liquid biopsies [50]. The following types of molecular entities have been reported as possibly relevant for oncology: (i) circulating tumour cells (CTCs) that can be assessed at the DNA, RNA and protein level; of note CTCs offer opportunities for functional assessment, (ii) cell-free DNA (cfDNA), alternatively called circulating tumour DNA (ctDNA), referring to DNA fragments shed to the circulation by cancer cells, (iii) tumour-educated blood platelets, which have been shown to be subjected to changes at the RNA level through their interaction with cancer cells [51], and (iv) microvesicles/exosomes, corresponding to extracellular vesicles, carrying protein- and nucleic acid-content that corresponds to biological messages transmitted from tumour cells to recipient normal cells [52, 53]. To the present day, CTCs and ctDNA are the most extensively studied types of liquid biopsies that could impact the following aspects of oncology:

### A. Cancer screening

In a study of lung cancer-free subject, CTCs were assessed in patients with chronic obstructive pulmonary disease (COPD) and subjects without this condition but matched for smoking habits [54]. There were five patients with COPD (3%) tested positive for CTCs and none from the control group; the annual CT surveillance detected lung parenchyma nodules 1–4 years following the CTCs detection, resulting in diagnosis of early-stage lung cancer [54]. Such results have not yet been reported for breast cancer; however, it could be envisioned that early CTCs’ detection could complement breast cancer screening for subjects at high risk. Emerging powerful technologies of ctDNA detection with increasing sensitivity could be also assessed as potential tools of personalised cancer screening [55].

### B. Monitoring of minimal residual disease

The assessment of minimal residual disease is a well-established clinical practice in the management of patients with leukaemia [56], but not in solid tumours; therapeutic decisions in the latter ones are taken on the basis of assessment of the primary tumour and there is no measurable parameter to inform about either response to treatment or occurrence of disease recurrence. The assessment of either ctDNA or CTCs could be possibly useful for the monitoring of minimal residual disease. In a prospectively conducted study, CTCs were assessed using the CellSearch® System evaluated patients with operable breast cancer before the onset of adjuvant chemotherapy administration and after chemotherapy [57]. Prior to adjuvant treatment, 21.5% of the patients were tested positive for CTCs, whereas after chemotherapy this percentage reached 19.6%; there was no correlation of standard clinico-pathologic risk factors and the detection of CTCs. CTCs detection was found to be an independent detrimental prognostic factor, indicating that CTCs could be a measurable parameter assessing minimal residual disease in patients with early-stage breast cancer.

A promising implementation of liquid biopsies would be to guide adjuvant treatment selection and monitor its therapeutic effect; such preliminary results have been generated through the assessment of disseminated tumour cells (DTCs) in the bone marrow of patients with early breast cancer [58].

### C. Guidance of treatment selection

The ultimate breast cancer medicine personalisation would occur through a ‘real-time’ monitoring of treatment activity, as well as a personalised treatment selection to target therapeutic vulnerabilities at the individual patient level. Theoretically, liquid biopsies could serve both purposes. In the classical paradigm, monitoring of treatment activity is performed through conventional image analysis and/or functional imaging assessments, complemented by clinical examination, in particular for patients with advanced disease; such objective read-outs to monitor treatment activity are lacking for patients with primary disease. This means practically that a patient must receive several cycles of treatment prior to the monitoring of the respective effect; for some patients, this means that the futility of a therapeutic strategy will be identified only retrospectively.

## Conclusions

Accumulation of knowledge over time has changed breast cancer from an incurable condition to a range of different diseases with specific molecular aberrations, clinical behaviours, and patterns of response to systemic treatments. These improvements are a huge achievement for humanity and have been accomplished over time with the contributions of many bright and passionate individuals. To continue this legacy, and take it to a new level of excellence, efforts in further understanding breast cancer biology and its interactions with the immune system and the microenvironment are being conducted. Prognostic and predictive genetic signatures are already a reality in breast cancer management and are being further refined. Liquid biopsy strategies are in current use for other indications and in research for breast cancer, in several settings. Refining our understanding of disease mechanisms and molecular characteristics is key to improving drug development and treatment approaches.

## Disclosures

The authors have declared no conflicts of interest related to the present manuscript.

## Figures and Tables

**Table 1.** Summary of main characteristics of the intrinsic subtypes of breast cancer.

	Luminal A	Luminal B	HER2-enriched	Basal like
**IHC Surrogate**	**ER(+) and/or PR(+), HER2(−), Ki67 < 14%** (St Gallen)	**ER(+) and/or PR(+), HER2(−), Ki67 ≥ 14% **(St Gallen)	**ER(±), PR(±), HER2(+)** (St Gallen)	**ER(−), PR(−), HER2(−)** (St Gallen)
**Prognosis**	Good	Intermediate	Poor	Poor
**Treatment vulnerability**	Endocrine treatment	Endocrine treatment + Cytotoxic chemotherapy	HER2 blockade	Cytotoxic chemotherapy
**Recurrent CNAs:** **Increased copy number** **Decreased copy number** **High-level amplification**	1q, 16p 16q 8p11-12, 11q13-14, 12q13-14, 17q11-12, 17q21-24, 20q13	1q, 8q, 17q, 20q 1p, 8p, 13q, 16q, 17p, 22q 8p11-12, 8q, 11q13-14	1q, 7p, 8q, 16p, 20q 1p, 8p, 13q, 18q 17q	3q, 8q, 10p 3p, 4p, 4q, 5q, 12q, 13q, 14q, 15q Rare
**Recurrent gene mutations (Top-5)**	*PIK3CA* (45%) *GATA3* (14%) *MAP3K1* (13%) *TP53* (12%) *CDH1* (9%)	*TP53* (29%) *PIK3CA* (29%) *GATA3* (15%) *MLL3* (6%) *MAP3K1* (5%)	*TP53* (72%) *PIK3CA* (39%) *MLL3* (7%) *AFF2* (5%) *PTPN22* (5%)	*TP53* (80%) *PIK3CA* (9%) *MLL3* (5%) *RB1* (4%) *AFF2* (4%)

**Table 2.** Summary of commercially available, first-generation, multigene prognosticators in breast cancer.

	Endopredict	MammaPrint	MapQuant DX	Oncotype DX	PAM50	Theros
**Number of Genes assessed**	8	70	97	21	50	2
**Types of tumour material**	Fresh-frozen	FFPE	Fresh-frozen	FFPE	FFPE	FFPE
**Technique**	qRT-PCR	DNA microarrays	DNA microarrays	qRT-PCR	nCounter	qRT-PCR
**BC subtype indication**	ER+/HER2-BC treated with adjuvant ET	Stage I or II BC, LN-, T ≤ 5 cm	ER+ Grade 2 BC, treated with adjuvant ET	ER+ BC treated with adjuvant ET and/or CT	ER+ BC treated with adjuvant ET	ER+ BC treated with adjuvant ET
**Prospective clinical evidence**	No	Yes (MINDACT)	No	Yes (TAILORx)	No	No
**Regulatory approval**	No	Yes (FDA)	No	No	No	No
**Company**	Sividon Diagnostics	Agendia	Ipsogen	Genomic Health	Nanostring Technologies	bioTheranostics

**Abbreviations:** BC: breast cancer, CT: chemotherapy, ER: oestrogen receptor, ET: endocrine therapy, FDA: food and drug administration, HER2: human epidermal growth factor receptor 2, LN: lymph node, MINDACT: microarray in node-negative and 1–3 positive lymph node disease may avoid chemotherapy, qRT-PCR: q-reverse transcription polymerase chain reaction, TAILORx: trial assigning individualised options for treatment (Rx)
